# Upstream Regulation of Development and Secondary Metabolism in *Aspergillus* Species

**DOI:** 10.3390/cells12010002

**Published:** 2022-12-20

**Authors:** Heungyun Moon, Kap-Hoon Han, Jae-Hyuk Yu

**Affiliations:** 1 Department of Bacteriology, University of Wisconsin-Madison, Madison, WI 53706, USA; 2 Department of Plant Pathology, University of Wisconsin-Madison, Madison, WI 53706, USA; 3 Department of Pharmaceutical Engineering, Woosuk University, Wanju 55338, Republic of Korea; 4 Department of Systems Biotechnology, KonKuk University, Seoul 05029, Republic of Korea

**Keywords:** *Aspergillus*, upstream regulators, G proteins, *velvet* regulators, NsdD, development, secondary metabolism

## Abstract

In filamentous fungal *Aspergillus* species, growth, development, and secondary metabolism are genetically programmed biological processes, which require precise coordination of diverse signaling elements, transcription factors (TFs), upstream and downstream regulators, and biosynthetic genes. For the last few decades, regulatory roles of these controllers in asexual/sexual development and primary/secondary metabolism of *Aspergillus* species have been extensively studied. Among a wide spectrum of regulators, a handful of global regulators govern upstream regulation of development and metabolism by directly and/or indirectly affecting the expression of various genes including TFs. In this review, with the model fungus *Aspergillus nidulans* as the central figure, we summarize the most well-studied main upstream regulators and their regulatory roles. Specifically, we present key functions of heterotrimeric G proteins and G protein-coupled receptors in signal transduction), the *velvet* family proteins governing development and metabolism, LaeA as a global regulator of secondary metabolism, and NsdD, a key GATA-type TF, affecting development and secondary metabolism and provide a snapshot of overall upstream regulatory processes underlying growth, development, and metabolism in *Aspergillus* fungi.

## 1. Introduction

Fungi are of crucial importance in human lives and natural environments, largely because of their various roles in medicine production, environmental recycling, food process, and agriculture. Due to their strong influence on humankind, researchers initiated an investigation on metabolic mutants of the filamentous fungus *Neurospora crassa* in 1941, which established the basis of genetics: the one gene, one protein hypothesis. Since then, fungi have served as model organisms for eukaryotic genetic studies advancing the knowledge of modern genetics [1]. Among multitudinous fungi, the genus *Aspergillus* is comprised of the most common and ubiquitous fungi, with more than 340 species being identified [2]. In addition to the prevalence, their relevance to the various areas in human health (*Aspergillus fumigatus*), industry (*Aspergillus niger* and *Aspergillus oryzae*), agriculture (*Aspergillus flavus*), and genetic research (*Aspergillus nidulans*) has drawn much attention from researchers leading to extensive genetic studies on *Aspergillus* species to understand the regulatory mechanisms of fungal growth, development, and metabolism.

*Aspergillus* fungi may undergo three different life cycles: asexual, sexual, and parasexual cycles [1]. Approximately only one-third of the identified *Aspergillus* species are known to have sexual development under appropriate conditions, and they primarily reproduce through asexual sporulation (conidiation) [3]. These developmental processes result in the formation of specialized structures, which generate and bear sexual (ascospores) or asexual (conidia) spores. In some *Aspergillus* species, it has been reported that developmental phases are tightly coordinated with the production of certain secondary metabolites including mycotoxins [4,5,6]. *Aspergillus* development is complex, precisely timed, and genetically programmed events involving specialized cellular differentiation, temporal and spatial regulation of gene expression, and intra- and inter-cellular communications, involving coordination of various TFs and regulators. Within this complex system, some regulators function at the upstream level bringing huge impacts on *Aspergillus* biology by controlling the expression of diverse downstream genes, which eventually leads to the alteration of developmental and metabolic processes. Not only TFs, but also any genes or molecules affecting gene expression are considered as upstream regulators [7].

**Figure 1:**
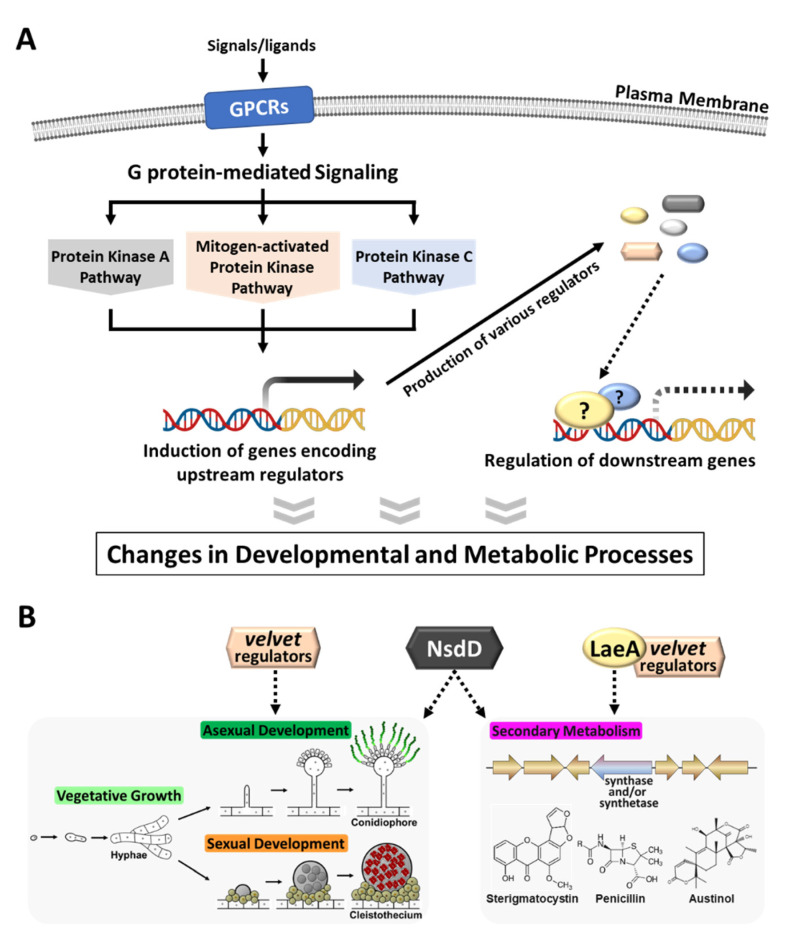
A schematic overview of upstream regulation in *Aspergillus* species. (**A**) The heterotrimeric G protein-mediated signaling pathway, initiated by binding of ligands or signals to the corresponding G-protein coupled receptors (GPCRs), relays external environmental signals inside the cell and initiates downstream signaling pathway(s): protein kinase A (PKA), mitogen-activated protein kinase (MAPK), and protein kinase C (PKC). As a result, the expression of genes encoding upstream regulators changed either up-regulated or down-regulated, which in turn affects expression levels of various downstream genes. Fungi efficiently control their growth, development, and metabolism through these signal transduction pathways. (**B**) Three different types of well-studied upstream regulations occurred by the *velvet* family regulators (developmental regulators), LaeA (a global regulator of secondary metabolism), and NsdD (a key regulator of asexual/sexual development). Solid line represents an activating role and dotted line represents a regulating role (either activating or repressing).

A large array of upstream regulators affecting conidiation, sexual development, and secondary metabolism in *Aspergillus* species have been identified and functionally studied [8,9,10,11,12,13,14]. In this review, we summarize a few different types of upstream regulators, known for their roles in main upstream regulation of *Aspergillus* biology (Figure 1): G proteins and G protein-coupled receptors (signal transduction), the *velvet* family proteins (development and metabolism), LaeA (secondary metabolism), and NsdD (development and secondary metabolism).

## 2. Heterotrimeric G Protein Signaling Governs Development and Metabolism

Sensing the external environment and adapting to surroundings are crucial for fungi to coordinate their growth and development accordingly. Fungi are responsive to environmental clues through the heterotrimeric G protein signaling pathway. G protein-coupled receptors (GPCRs) relay signals from the extracellular environment inside the cell by existing across a cell membrane. These receptors react to a large spectrum of external signals including light, hormones, neurotransmitters, cytokines, growth factors, cell adhesion molecules, and nutrients, such as sugars, amino acids, and nitrogen sources [15]. Binding to extracellular molecules causes a conformational change in GPCRs and this alteration then activates the interaction between the GPCR and G protein, which bound to C-terminus of the GPCR in cytosol. When the G protein becomes active by exchanging GDP with GTP, it can trigger the production of thousands of second messenger molecules, such as cyclic AMP (cAMP), diacylglycerol (DAG), and inositol 1, 4, 5-triphosphate (IP3), which initiate and coordinate further intracellular signaling pathways [16]. In fungi, G protein-mediated signaling pathways include the cAMP-dependent protein kinase (PKA) and the mitogen-activated protein kinase (MAPK) pathways. Through these sequences of events, fungi regulate their growth, development, morphogenesis, mating, metabolism, virulence, and mycotoxin biosynthesis according to the environments where they are situated [17,18].

### 2.1. G Protein-Coupled Receptors (GPCRs) 

G protein-coupled receptors (GPCRs) are transmembrane proteins and the largest class of cell surface receptors in fungi. GPCRs are plasma-membrane-localized proteins that communicate changes in the environment to intracellular heterotrimeric G proteins [16]. GPCRs contain seven transmembrane (7-TM) helices connected by intracellular and extracellular loops, with an extracellular amino-terminus (N-terminus) and the carboxyl-terminus (C-terminus) extending into the cytoplasm. Although the majority of GPCRs consist of 7-TM helices, some GPCRs are containing 5-TM or 6-TM helices, such as GprB, GprG, GprN, and NopA. According to the previous studies, *Aspergillus* GPCRs are classified into ten different classes. Eighteen GPCRs named GprA to GprS and NopA belong to Class I to IX, and Class X GPCRs are Pth11-like receptors, which promote fungal-plant pathogenic interactions in *A*. *nidulans* (Table 1). GprN was specifically identified in *A. nidulans,* and GprR and GprS were exclusively identified in *A*. *flavus*, which contain the regulator of G protein signaling (RGS) domain and PQ-loop repeat domain, respectively [19]. Despite their prevalence and fundamental roles in fungi, only a few GPCRs have been identified and functionally characterized. In recent years, the rapid development and evolution of Next-Generation Sequencing technology have boosted GPCR studies by enabling accessible large-scale whole genome sequencing, which led to figuring out putative GPCRs in genome based on structural similarities and putative activating ligands. These studies have revealed that the *A. nidulans* genome encodes 86 putative GPCRs, which can be divided into 16 GPCRs in 9 categories (Class I to IX) and 70 class X Pth11-like receptors [20,21,22,23]. The major producer of aflatoxins *A. flavus*’ genome encodes fifteen putative GPCRs in nine categories (Class I to IX), and Class X GPCRs still remain to be identified (reviewed in [19]). In the opportunistic human pathogen *A. fumigatus*, the genome encodes fifteen putative classical GPCRs (Class I to IX), yet only five of them (GprC, GprD, GprK, GprM, and GprJ) have been characterized [24,25,26]. Functional studies on GPCRs have unveiled that they play significant roles in overall fungal biology relating to nutrient sensing, fungal development, pheromone response, fruiting body formation, mycotoxin production, and pathogenesis. According to functional characteristics, GPCRs can be categorized into 10 groups: pheromone (classes I and II), carbon (III), nitrogen (IV), cAMP receptor-like (V), RGS (Regulator of G protein signaling, VI), MG00532-like (VII), mPR-like (VIII), microbial opsin (IX), and Pth11-like (X) receptors. 

The pheromone receptors were first identified in *Saccharomyces cerevisiae*. Two different pheromone receptors, Ste2p (α-factor receptor) and Ste3p (a-factor receptor), presented in the cell membranes of opposite haploid mating types (MATα and MATa). When yeast cells are exposed to the pheromone secreted by the opposite mating type, their pheromone receptors are activated and initiate G protein-mediated signaling pathway leading to the eventual fusion with the mating partner [27]. Carbon-sensing receptors regulate the response to carbon sources in fungi. In the filamentous fungus *N*. *crassa*, GPR-4 (G-protein coupled receptor 4) physically interacts with the Gα (GNA-1) to regulate carbon source-dependent growth and development. The gpr-4 null mutants displayed less mass accumulation compared to the WT in carbon-limited conditions and no transient increase in cAMP levels upon a nutrient shift from carbon-limited to glucose-rich media, which was normally observed in WT [28]. Nitrogen-sensing receptors act in a very similar way to carbon-sensing ones. In *Schizosaccharomyces pombe*, the Stm1 receptor, coupling with the Gα2 protein, is required for proper recognition of nitrogen starvation signals. Overexpression of Stm1 led to the inhibition of vegetative growth and the decrease in intracellular cAMP levels even under nutrient-rich conditions [29]. The cAMP receptors (cARs) were firstly identified in *Dictyostelium discoideum* and then the sequences of cARs were used to predict cAMP receptor-like GPCRs (Crls) in *D. discoideum* and likely fungal cAMP receptor genes. The cARs are known to play significant roles during divergent developmental stages and in distinct subsets of developing cells within multicellular aggregates by interacting with secreted cAMP [30,31,32]. The *N. crassa* GPR-1, distantly related to the four cAMP receptors (cAR1 to cAR4) and three cAMP receptor-like GPCRs (CrlA to CrlC), was the first cAMP receptor-like GPCR characterized in ascomycete fungi. In *N. crassa*, GPR-1 is localized in female reproductive structures and regulates female sexual development [33]. The GPCR containing RGS domain was firstly discovered in *Arabidopsis thaliana*. The *A. thaliana* RGS, AtRGS1 protein negatively regulates the Gpa1 Gα subunit affecting cellular proliferation. Canonical GPCRs cause the conformational change of G protein triggering the GDP-GTP exchange, but instead, AtRGS1 interacts with the active Gα subunit resulting in hydrolysis of GTP, which in turn deactivates the G protein [34]. This type of GPCR has been found in several species of filamentous fungi. In *Aspergillus* species, GprK containing both 7-TM and RGS domains is similar to AtRGS1 and involved in germination, development, and stress response [25]. The MG00532 group was represented by a protein with weak homology to rat growth hormone-releasing factor. The mPR-like class of GPCR includes proteins related to the human membrane progesterone receptors (mPRs), which mediate an array of rapid, cell surface-initiated progesterone actions in the reproductive system involving activation of intracellular signaling pathways [35,36]. The microbial opsins are a class of retinal-binding proteins with seven membrane-spanning domains that form rhodopsins by interacting with the retinal and function as light-responsive ion pumps or sensory receptors. The NOP-1 protein of *N. crassa*, closely related to archaeal opsins, was the first opsin characterized in filamentous fungi and known to bind all-*trans* retinal by using a Schiff base linkage and play a role in *N. crassa* photobiology [37]. The PTH11 protein was first discovered in the plant pathogenic fungus *Magnaporthe oryzae* and identified as an activator of appressorium differentiation in response to inductive surfaces. The aberration of the *pth11* gene in *M. oryzae* led to the defect in pathogenicity [22]. Although a large number of Pth11-like GPCRs have been predicted, in *Aspergillus,* their exact functions remain heavily unknown [19,38]. 

In the presence of extracellular signals, corresponding GPCRs recognize molecules and relay the signal inside the cell. The recognition of external cues by GPCRs provokes the conformational change of G protein, which in turn initiates the G protein signaling pathways including the cAMP-dependent protein kinase (PKA) and the mitogen-activated protein kinase (MAPK) pathways.

**Table 1:** G protein-coupled receptors in *Aspergillus* species.

Gene	Class	TM No.	Conserved Domains (Note)	Ref
***gprA***	I	7-TM	Ste2; alpha-pheromone receptor (*S. cerevisiae* pheromone receptor)	[39]
***gprB***	II	5-TM	Ste3; a-pheromone receptor (*S. cerevisiae* pheromone receptor)	[39]
***gprC***	III	7-TM	Git3; Gpa2 C-term; Family 1-like (*S. pombe* glucose receptor)	[40]
***gprD***	III	7-TM	Git3; Gpa2 C-term (*S. pombe* glucose receptor)	[40]
***gprE***	III	7-TM	Git3; Gpa2 C-term (*S. pombe* glucose receptor)	[40]
***gprF***	IV	7-TM	PQ-loop repeat (*S. pombe* nitrogen sensor)	[40]
***gprG***	IV	5-TM	PQ-loop repeat (*S. pombe* nitrogen sensor)	[40]
***gprJ***	IV	7-TM	PQ-loop repeat (*S. pombe* nitrogen sensor)	[38]
***gprS***	IV	7-TM	PQ loop repeat (*S. pombe* nitrogen sensor)	[19]
***gprH***	V	7-TM	Secretin-like; cAR (*D. discoideum*); Family 2-like (signal through cAMP pathways)	[40]
***gprI***	V	7-TM	cAR (*D. discoideum*); Family 2-like (signal through cAMP pathways)	[40]
***gprK***	VI	7-TM	RGS (regulator of G protein signaling)	[38]
***gprR***	VI	7-TM	RGS (regulator of G protein signaling)	[19]
***gprM***	VII	7-TM	MG00532-like	[38]
***gprN***	VII	6-TM	MG00532-like	[38]
***gprO***	VIII	7-TM	mPR-like; Hemolysin-III related (broad range of ligands)	[38]
***gprP***	VIII	7-TM	mPR-like; Hemolysin-III related (broad range of ligands)	[38]
***gprQ***	VIII	5-TM	mPR-like; Hemolysin-III related (broad range of ligands)	[38]
***nopA***	IX	6-TM	Bacterial rhodopsin-like (photoreactive)	[38]
***pth11*** **-like**	X	7-TM	Pth11-related group	[38]

### 2.2. G Protein-Mediated Signaling Pathway

**Figure 2:**
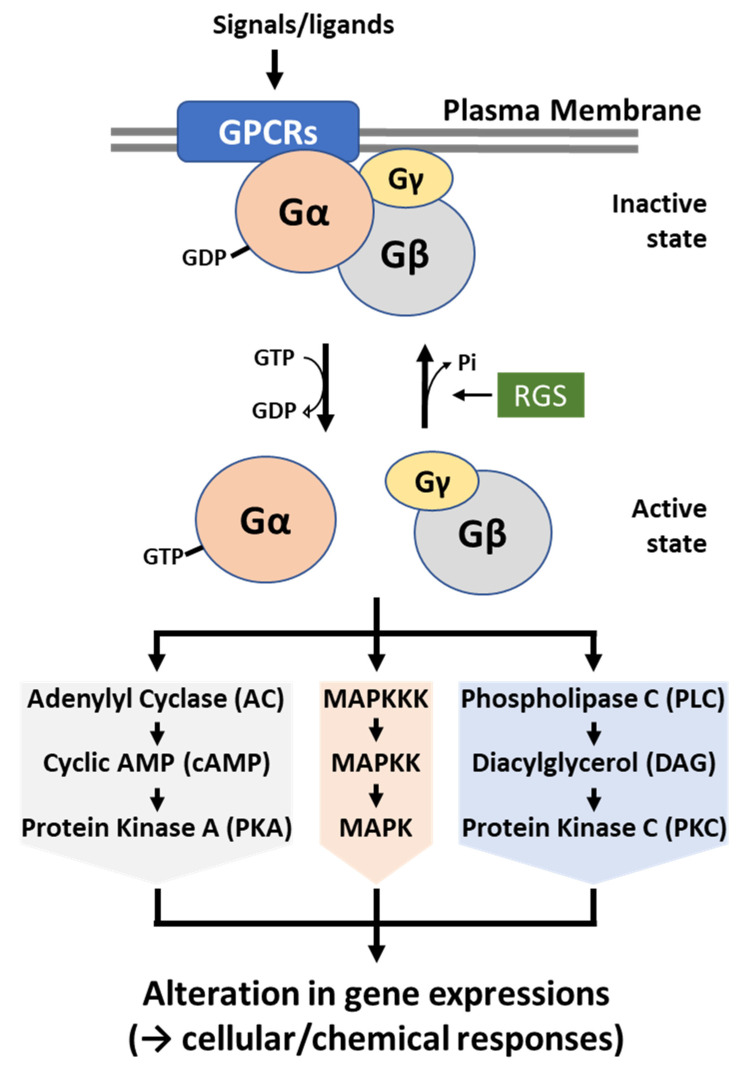
A schematic diagram of heterotrimeric G protein-mediated signaling pathway. Upon the recognition of external ligands, a G protein-coupled receptor (GPCR) is sensitized and interacts with a nearby G protein. Then, G proteins become active by the interaction with GTP and initiate downstream signaling pathway(s): PKA, MAPK, and PKC. This signal transduction results in the differential expression of genes involved in growth, development, morphogenesis, mating, metabolism, virulence, and mycotoxin biosynthesis. The activated G proteins are then negatively controlled by regulators of G protein signaling (RGS) and become inactive again.

Heterotrimeric guanine nucleotide-binding proteins (G proteins), consisting of alpha, beta, and gamma subunits, have been characterized in diverse eukaryotic organisms demonstrating their pivotal roles in major signal transduction pathways during the responses of cells to extracellular stimuli. The G proteins, present in all eukaryotic cells, control metabolic and developmental pathways (reviewed in [41]). In filamentous fungi, the first G proteins, particularly α subunits, were discovered in *N. crassa* in the early 1990s. Thereafter, the G proteins FadA (α subunit), SfaD (β subunit), and GpgA (γ subunit) were identified in *A. nidulans* [42,43,44]. The inactive G protein heterotrimer is composed of α, β, and γ subunits, whereas the α and γ subunits are associated with the plasma membrane. Upon binding of specific ligands, GPCRs experience a conformational change, then physically interact with heterotrimeric G proteins. This physical interaction results in an exchange of GTP for GDP on the Gα subunit, which in turn leads to the dissociation of the heterotrimer into the GTP-Gα subunit and the Gβγ heterodimer. Once dissociated, the GTP-Gα and the Gβγ become active so that GTP-Gα, Gβγ, or both moieties can relay and amplify signals by regulating activities of downstream effector proteins in divergent signal transduction pathways. Then, RGS proteins interact with an activated GTP-Gα subunit and increase its intrinsic GTPase activity. The GTP hydrolysis enables GDP-Gα subunit to reassociate with Gβγ heterodimer and plasma membrane, becoming into the inactive form of heterotrimeric G proteins again. In fungi, G protein-mediated signaling pathway is transmitted through one or more of the following pathways: (1) cAMP-dependent protein kinase (PKA), (2) mitogen-activated protein kinase (MAPK), and (3) Ca^2+^- and DAG-dependent protein kinase C (PKC) (Figure 2) [45,46].

The *Aspergillus* species possess three distinct groups of Gα proteins. Each group of Gα was assigned according to the amino acid sequence similarity with the *N. crassa* Gα proteins; Gna-1 (group I), Gna-2 (group II), and Gna-3 (group III). The group I Gα proteins possess a consensus sequence for myristoylation (MGXXXS) at the N-terminus and a site for ADP-ribosylation by pertussis toxin (CAAX) at the C-terminus. Most well-characterized filamentous fungi are known to possess a single group I Gα protein and its function has been well elucidated. The group III Gα proteins are also highly conserved and possess a myristoylation at the N-terminus. They are known to positively influence cAMP levels. However, the functions of group II Gα proteins are not as obvious as group I and III Gα proteins (reviewed in [20]). 

In *A. nidulans*, the first characterized group I Gα subunit was FadA, showing 93% identity of AA sequence to *N. crassa* Gna-1 and, thereafter, GanA (group II) and GanB (group III) had been identified. FadA (fluffy autolytic dominant) was initially investigated for the fluffy autolytic phenotype, which was attributed to an uncontrolled vegetative growth followed by autolysis [42]. The dominant activating mutations on FadA resulted in the expression of the fluffy autolytic phenotype and the inhibition of mycotoxin production, especially sterigmatocystin (ST), while dominant interfering FadA mutants displayed reduced vegetative growth, enhanced asexual sporulation, and precocious ST production [42,47]. Constitutively active dominant FadA mutants were presumed to maintain a longer period of the activated state of FadA-GTP due to the decreased intrinsic GTPase activity. Taken together, these results indicated that activated GTP-FadA (Gα) mediates signaling via cAMP-dependent PKA that promotes vegetative growth, which in turn suppresses asexual sporulation, sexual development, and mycotoxin production in *Aspergillus*. 

The role of the group II Gα subunit, GanA has been well studied in *A. fumigatus*. GanA shares 46.3% and 44.3% identity with GpaA (group I, the homolog of FadA) and GpaB (group III, the homolog of GanB) in *A. fumigatus*, respectively. The mRNA level of *ganA* was highly expressed at both early (6 h) and later (48 h) time of asexual development and the deletion of the *ganA* gene resulted in faster germination, but decreased radial growth compared to those of WT on solid media; however, it did not show any significant impact on asexual development, unlike other Gα proteins. In addition, the Δ*ganA* strain displayed reduced mRNA level of gliotoxin biosynthesis transcription factor *gliZ* and decreased GT production compared to those of WT as well. Interestingly, the *ganA* null mutant exhibited the highest activity of PKA in conidia and PKC in mycelia among Gα mutants [48]. These results demonstrated that GanA plays important roles in vegetative growth, asexual development, and mycotoxin production through PKA or PKC signaling pathway in a similar, but slightly different way from other groups of Gα proteins. 

**Figure 3:**
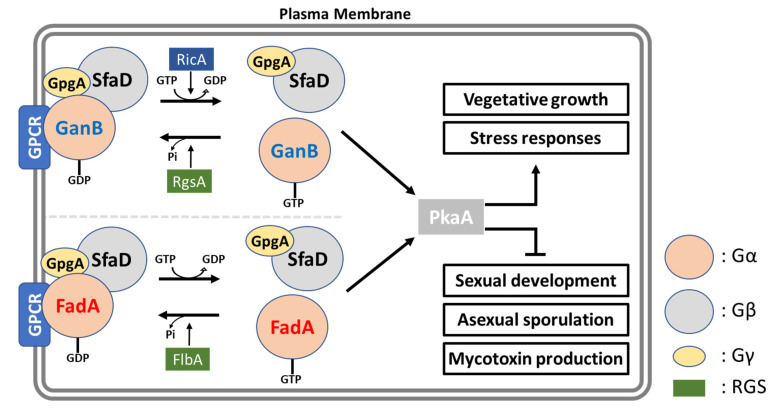
Heterotrimeric G protein-mediated signaling pathway in *A. nidulans*. In *A. nidulans*, two Gα subunits (FadA and GanB) regulate vegetative growth, development, and mycotoxin production via PKA pathway when they are in the activated state. The regulators of G protein signaling (RGS) FlbA and RgsA turn activated FadA and GanB back into the inactive state, respectively. RicA is a GDP/GTP exchange factor involved in the activation of G protein subunits.

The group III Gα protein (GanB) is functionally well characterized in *A. nidulans* by Chang et al. [49] and Lafon et al. [50]. They revealed that GanB positively regulates conidial germination but inhibits asexual sporulation by mediating a rapid and transient increase in cAMP levels in response to the presence of extracellular glucose during the early phase of germination. Moreover, Lafon et al. [50] elucidated that GanB (Gα) and SfaD::GpgA (Gβγ) form a heterotrimeric complex. Collectively, G protein α subunits in *Aspergillus* mediate signaling that promotes vegetative growth and stress responses, which in turn inhibit fungal development and mycotoxin production (Figure 3).

Most filamentous fungi are predicted to have a highly conserved single Gβ subunit (from 66 to 92% identical with *N. crassa* Gnb-1) and Gγ subunit (from 39 to 92% identical with *N. crassa* Gng-1) [20]. Previous studies on the Gβ and Gγ mutations have shown that mutational inactivation of genes encoding these proteins affected vegetative growth, conidiation, and sexual development in filamentous fungi [51,52]. Particularly, Krystofova and Borkovich [33] demonstrated that the *gng-1* and *gnb-1* loss-of-function mutations displayed similar phenotypes, such as female sterility, defective conidiation, low levels of intracellular cAMP, and a severe reduction in Gα protein levels in *N. crassa*. In addition, they proposed that Gng-1 (Gγ) physically interacts with Gnb-1 (Gβ) and forms the Gnb-1:Gng-1 (Gβγ) heterodimer during signaling pathways. In the genus *Aspergillus*, the Gβ subunit SfaD and Gγ subunit GpgA were identified and well characterized. Rosén et al. [43] isolated SfaD composed of 352 AA that shares 86% identity with *N. crassa* Gnb-1 and revealed that SfaD has a conserved Trp-Asp sequence, which is known as a WD40 domain. They revealed that SfaD plays crucial roles in vegetative growth, conidial sporulation, sexual development, and ST production in *A. nidulans*. Moreover, Seo et al. [44] identified the Gγ subunit GpgA, which consists of 90 AA that shows 65% identity with *N. crassa* Gng-1. The *gpgA* loss-of-function mutation exhibited reduced vegetative growth, delayed conidiation, and no sexual fruiting body formation, similar to Δ*sfaD* mutants. Later, Lafon et al. [50] revealed that the SfaD:GpgA (Gβγ) heterodimer is crucial for the proper activation of GanB (Gα), while GanB plays a primary role in the PKA signaling pathway in response to glucose. 

Timely modulation of G protein-mediated signaling pathways is the key for fungi in sensing and responding to internal/external signals and various stress conditions. Upon the recognition of extracellular signals, cells need to activate G proteins immediately, so they can translate diverse incoming signals into corresponding cellular responses opportunely. However, the prolonged activated state of GTP-Gα can cause various defects in fungal development and metabolism. Thus, the neutralization of activated GTP-Gα into the inactive form on time is as significant as the activation. These tight upstream regulations play crucial roles in vegetative growth, development, mycotoxin production, and virulence in fungi. There are three different types of regulators present in *Aspergillus* species: phosducin-like proteins (PhLPs), regulators of G protein signaling (RGSs), and a GDP/GTP exchange factor (RicA).

Phosducin-like proteins are a group of evolutionarily conserved positive regulators of Gβγ heterodimer function. PhLPs act as molecular chaperones during Gβγ assembly by stabilizing the nascent Gβ subunit until it associates with the Gγ protein [53,54]. In *A. nidulans*, three potential PhLPs (PhnA, PhnB, and PhnC) were identified based on the AA sequence similarity with Bdm-1, which is a known fungal Gβγ activator in the chestnut blight fungus *Cryphonectria parasitica* [55]. Among them, the function of PhnA was firstly investigated by Seo and Yu [56] due to its highest similarity with Bdm-1. Seo and Yu [56] revealed that PhnA is required for proper SfaD functionality, sexual reproduction, and mycotoxin biosynthesis showing consistent results with the roles of SfaD::GpgA heterodimer. 

RGSs are a group of proteins containing a conserved ∼130 AA RGS box, which physically interact with an activated GTP-Gα and accelerate the intrinsic GTPase activity of the Gα subunit, resulting in the attenuation of G protein-mediated signaling pathways [46,57]. In *Aspergillus*, several RGSs have been identified including FlbA, RgsA, RgsB, RgsC, GprK, and Rax1. Among them, FlbA and RgsA are the most well-characterized RGSs (Figure 3). The FlbA consists of 719 amino acids containing 1 RGS box and 2 DEP (Dishevelled, EGL-10, and Pleckstrin) domains. The DEP is a globular protein domain of ~80 AA commonly found in proteins involved in G-protein signaling, however, the repeated pattern of DEP is only observed in fungi [58]. Along with the GTPase-activating RGS domain, the DEP domain may play a role in guiding RGS proteins to the Golgi and plasma membranes [59]. The *flbA* loss-of-function exhibited the fluffy-autolytic phenotype, which was observed in activating dominant FadA (Gα) mutants. In addition, the *fadA* deletion mutants did not display the fluffy-autolytic phenotype caused by Δ*flbA* and restored asexual development and mycotoxin production. The primary role of FlbA is to attenuate G protein-mediated signaling by deactivating GTP-FadA (group I Gα) protein, whereas FadA, SfaD, and GpgA constitute the major G protein heterotrimer modulating growth, development, and secondary metabolism in *A. nidulans* [42,47]. The RgsA consists of 362 AA containing one RGS box in the N-terminal region. Unlike FlbA regulating group I Gα subunit, RgsA negatively regulates GanB (group III Gα) signaling, which promotes stress responses via the PKA pathway resulting in the inhibition of asexual development [58]. 

The GDP/GTP exchange factor RicA is recently discovered compared to other regulators of G proteins in *Aspergillus*. The *ricA* deletion mutants displayed severely reduced colony growth, and a total absence of asexual sporulation and sexual development in *A. nidulans* [60]. Kwon et al. [60] introduced the *A. fumigatus ricA* gene (Af*ricA*) into *A. nidulans* Δ*ricA* and found that the overexpression of Af*ricA* in the ΔAn*ricA* mutant partially restored colony growth and asexual development. In addition, they revealed that the removal of only *rgsA*, not *sfgA*, *flbA*, *rgsB*, or *rgsC*, restored vegetative growth and conidiation in ΔAn*ricA* and that RicA can physically interact with GanB (Gα) in vitro in yeast. These results led Kwon et al. [60] to conclude that RicA primarily activates the GanB initiating PKA signaling cascade in *A. nidulans*. 

## 3. The *velvet* Regulators and the Global Regulator of Secondary Metabolism LaeA

In filamentous fungi, fungal development, and secondary metabolism are intimately associated via the activities of the fungal-specific *velvet* family regulatory proteins and the global regulator of secondary metabolism LaeA. The *velvet* regulators form various complexes playing divergent roles in fungal development. The VosA-VelB heterodimer inhibits conidial germination, but positively regulates trehalose synthesis and β-glucan biogenesis. Moreover, VeA bridges VelB and LaeA to form the VelB-VeA-LaeA (*velvet*) heterotrimeric complex in the absence of light and this *velvet* complex controls not only secondary metabolism, but also the formation of Hülle cells, which nurse the nascent sexual fruiting bodies. This section summarizes the functions of *velvet* proteins and LaeA in *Aspergillus*.

### 3.1. The velvet Family Regulators

The first study of the *velvet* family regulators was conducted on the *velvetA1* mutant (renamed as *veA1* afterward). The colony of this mutant showed a flat and velvety appearance regardless of the presence or absence of light [61]. The functions of the *veA* gene have been extensively characterized in *Aspergillus* species. The *veA* gene is necessary for proper sexual development and secondary metabolism in *Aspergillus* [62,63,64,65,66,67,68]. In 2007, a major advancement in the *velvet* family study was made by Ni and Yu [69]. They demonstrated that the novel regulator VosA (viability of spores) governs sporogenesis and trehalose biogenesis, which in turn determines the viability of spores in *A. nidulans*. In addition, they discovered two other proteins that are similar to VosA; they named them VelB (velvet-like protein) and VelC. These four proteins were designated as the *velvet* family of regulators [69]. The *velvet* family proteins are highly conserved in Aspergilli and they all share a fungi-specific and highly conserved *velvet* domain, which consists of approximately 170–300 AA sequences with three conserved motifs [9].

### 3.2. The velvet Regulators in A. nidulans

**Figure 4:**
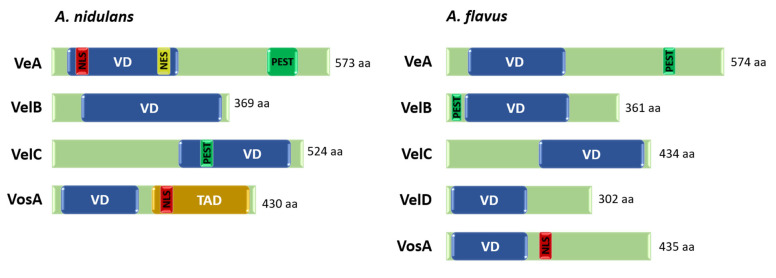
Domain architecture of the *velvet* regulators in *A. nidulans* and *A. flavus*. VD: *velvet* domain, NLS: Nuclear localization signal, NES: nuclear export signal, PEST: Proline (P)-, glutamic acid (E)-, serine (S)-, and threonine (T)-rich sequence, TAD: transcription activation domain, aa: amino acids.

VeA was the first member of the *velvet* family regulators identified in the 1960s [70]. VeA is composed of 573 amino acids containing the *velvet* domain in the N-terminal region (Figure 4). In addition, in the N-terminal region of this regulator, a potential nuclear localization signal (NLS) and nuclear export signal (NES) domains were found, suggesting roles in the nuclear localization of VeA [71]. Moreover, VeA contains a putative PEST (proline (P)-, glutamic acid (E)-, serine (S)-, and threonine (T)-rich) sequence in the C-terminal region [62], which is commonly found in rapidly degraded proteins [72]. VeA is a key light-dependent developmental regulator that positively regulates sexual development, which in turn suppresses asexual sporulation in *A. nidulans* [73,74]. The *veA* loss-of-function mutations resulted in the complete absence of sexual fruiting body formation, even under sexual development-promoting conditions, whereas the overexpression of *veA* enhanced the production of cleistothecia but inhibited asexual sporulation [62]. Furthermore, VeA is known to play crucial roles in secondary metabolism. VeA acts as an activator on sterigmatocystin production but inhibits penicillin biosynthesis [63,75]. Underlying the significant roles of VeA in fungal development and secondary metabolism, the nuclear localization of VeA is a vital factor. The VeA protein is constitutively expressed during the life cycle of *A. nidulans* but is mostly found in the cytoplasm under the presence of light [62,71,76]. On the other hand, in the dark, VeA enters the nucleus, forms VelB-VeA-LaeA heterotrimeric complex, and controls sexual development and mycotoxin production [71,77].

VosA consists of 430 AA containing the *velvet*, NLS, and potential TAD (transcription activation) domains, indicating that it may function as a transcription factor. VosA protein is expressed during vegetative growth and the early stage of asexual and sexual development, however, primarily localized in the nucleus of mature conidia. Interestingly, the expression of *vosA* is regulated by AbaA. In phialides, AbaA binds to the promoter region of *vosA* and induces the accumulation of *vosA* mRNA in conidia during the late phase of asexual development [67,69]. VosA is a key regulator of conidiation and sexual development. The *vosA* null mutants produced asexual developmental structures (conidiophores) in the liquid submerged culture, where the wild type solely undergoes vegetative growth and produced fewer numbers of cleistothecia compared to the WT. In addition, the deletion of *vosA* resulted in the accumulation of high mRNA levels of the *brlA* gene, which is a key initiative factor of conidiation, indicating VosA is a key negative regulator of *brlA* [69]. Moreover, VosA controls various biological processes including conidia wall integrity, spore viability, conidial germination, and focal trehalose biogenesis [78,79]. 

VelB is a 369-AA protein containing the *velvet* domain covering the entire protein. The *velB* gene is mostly expressed during the life cycle, but particularly high levels of *velB* mRNA are observed during vegetative growth and in the late phases of asexual and sexual development. Similarly, VelB protein is detectable during entire vegetative growth and in early developmental stages. VelB has divergent functions regulating vegetative growth, development, and secondary metabolism. VelB negatively regulates conidial germination but acts as an activator of asexual development. The *velB* deletion mutants showed increased conidial germination rates yet exhibited a reduced conidia production and decreased expression levels of asexual development-related genes, such as *brlA* and *abaA*. In addition, the deletion of *velB* led to the enhanced production of brown pigments [67,77]. 

VelC is composed of 524 AA containing the *velvet* and PEST domains in the C-terminal region. Unlike other *velvet* family members, the mRNA of *velC* specifically accumulates during the early phase of sexual development. The aberration of the *velC* gene led to the slightly enhanced conidia production and increased the mRNA levels of all three central regulatory genes of conidiation, *brlA*, *abaA*, and *wetA,* regardless of the presence or absence of light. In addition, the deletion of *velC* resulted in a decreased production of sexual fruiting bodies, while overexpression of this gene led to increased production of cleistothecia but a decreased number of conidia. These suggest that VelC plays a role in the activation of sexual development [9]. 

**Figure 5:**
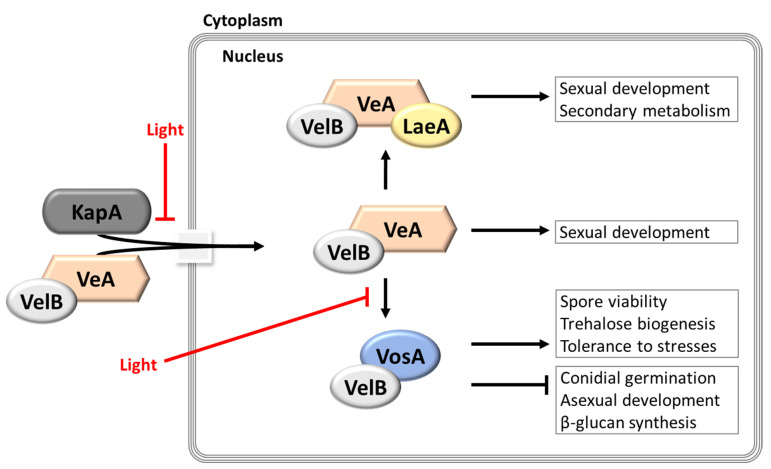
The functions of *velvet* family proteins in *Aspergillus* species. This model is adopted and modified from [82]. VeA enters the nucleus together with VelB and the importin-α KapA in the dark. In the nucleus, *velvet* family proteins and LaeA can form three different complexes depending on the presence or absence of light: VelB-VosA, VelB-VeA, and VelB-VeA-LaeA. These complexes regulate different biological processes.

Individual *velvet* protein plays multifunctional roles in *Aspergillus* development and metabolism; however, the most significant trait of *velvet* proteins is that they interact with partner proteins including themselves, and form complexes in multiple combinations, which govern the various processes of fungal biology. The formation of *velvet* protein complexes occurs in a cell type- and/or timing-specific manners. Among all different *velvet* protein complexes, the three most extensively studied complexes, VosA-VelB, VelB-VeA, and VelB-VeA-LaeA, are discussed here (Figure 5). During germination, the VosA-VelB heterodimer inhibits conidial germination rates. Moreover, the VosA-VelB heterodimer controls spore viability, trehalose biogenesis, β-glucan synthesis, and tolerance of conidia to various stresses, such as heat and oxidative stress. The VelB-VeA complex is a key participant in sexual development. Although the molecular mechanism of the VelB-VeA heterodimer formation has not been clearly identified yet, the VelB-VeA complex plays an important role in sexual development as an activator. Furthermore, as VeA bridges between the VelB-VeA heterodimer and LaeA, the VelB-VeA complex interacts with LaeA and forms the VelB-VeA-LaeA heterotrimeric complex in the nucleus. This VelB-VeA-LaeA complex regulates sterigmatocystin production and sexual development in the dark. In addition, it controls the expression of secondary metabolism-related genes at transcriptional or epigenetic levels [77,80,81].

### 3.3. The velvet Regulators in Other Aspergillus Species

Most *Aspergillus* species have four *velvet* family regulators (VeA, VelB, VelC, and VosA), but a recent study newly identified the fifth member of the *velvet* family, VelD, in *A. flavus* [83]. Although the *velvet* family proteins are highly conserved in Aspergilli, their functions might have been divergent depending on the species. In the plant pathogenic and mycotoxigenic fungus *A. flavus*, the VeA protein was first identified among the *velvet* regulators and revealed to regulate asexual and sexual development and mycotoxin production including the most carcinogenic mycotoxin aflatoxin [84]. The deletion of the *veA* gene decreased the production of conidia and completely blocked sclerotia formation. The *veA* null mutant was also unable to produce aflatoxins and aflatrem. Moreover, VeA regulates the mRNA expression of genes associated with various secondary metabolite production, such as aflatoxin, aflatrem, and asparasone [84,85]. Similar to *A. nidulans* VeA, *A. flavus* VeA interacts with VelB and LaeA to form the VelB-VeA-LaeA complex regulating sclerotia formation and aflatoxin production [68]. Of note, the deletion of *veA* or *velB*, but not *laeA*, resulted in the impaired conidiation, implying the positive regulation of VeA-VelB on asexual development in *A. flavus* [68]. The VosA-VelB heterodimer is required for proper trehalose biosynthesis and tolerance of conidia to various stresses. The newly identified VelD plays a role in aflatoxin production as the Δ*velD* mutant showed no aflatoxin production [83]. In the opportunistic human pathogenic fungus *A. fumigatus*, *velvet* family regulators except VelC are required for proper asexual development. The *veA*, *velB*, or *vosA* null mutants exhibited asexual development even in the liquid submerged culture, where only vegetative growth occurs for the WT and the accumulation of high *brlA* mRNA levels, indicating a repressive role of these *velvet* regulators in conidiation. In addition, VeA positively regulates the production of gliotoxin, which is known to inhibit the human immune response. Unlike *A. nidulans* and *A. flavus*, the roles of the VelB-VeA-LaeA complex in *A. fumigatus* are not clear yet; however, the cross-species complementation analysis suggests that the VelB-VeA-LaeA complex of *A. fumigatus* plays a similar role with those of *A. nidulans* in that the introduction of the *A. nidulans veA* gene into the *A. fumigatus* Δ*veA* restored the normal phenotypes in *A. fumigatus*. The VosA-VelB complex is necessary for spore viability, trehalose biosynthesis, and tolerance of conidia to UV and oxidative stresses [67].

### 3.4. LaeA, a Global Regulator of Secondary Metabolism

**Figure 6:**
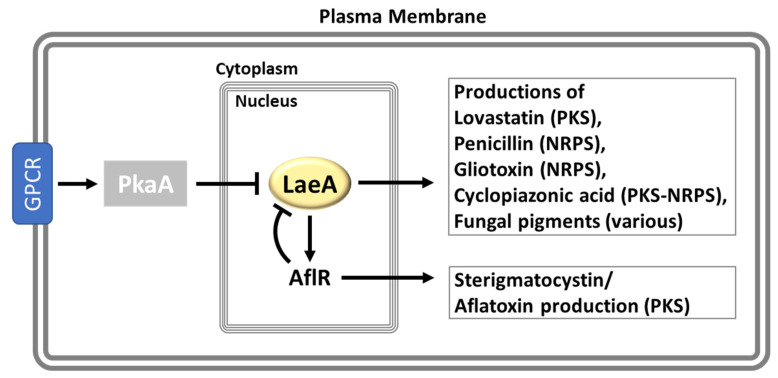
The functions of LaeA in *Aspergillus*. See the “LaeA, a Global Regulator of Secondary Metabolism” section.

Secondary metabolism is inseparable from fungal growth and development. Secondary metabolites have received much attention due to their broad spectrum of pharmaceutical and/or toxic properties: antibiotic, antiviral, antitumor, and immunosuppressive activities, and phytotoxic and mycotoxic activities [4,86,87]. Two decades ago, Butchko et al. [88] performed mutagenesis screening on 23 mutants that exhibited loss of sterigmatocystin (ST) production but normal asexual development in *A. nidulans* to reveal genes that are specific for the regulation of secondary metabolism. Thereafter, Bok and Keller investigated one of these mutants and identified a novel nuclear protein, LaeA, as a global regulator of secondary metabolism in *Aspergillus* (Figure 6) [87]. 

In *A. nidulans*, LaeA is required not only for the biosynthesis of a large array of secondary metabolites (SM) but also for the proper expression of corresponding SM biosynthetic gene clusters. The deletion of *laeA* inhibited the production of ST, the β-lactam antibiotics penicillin (PN), the anti-hypercholesterolaemic agent lovastatin (LOV), and the biosynthesis of mycelial pigments, which is a visually noticeable phenotype of Δ*laeA*. The *laeA* null mutant exhibited a near absent mRNA expression of the *aflR* and *stcU* genes encoding a transcription factor and a biosynthetic enzyme required for ST production. Furthermore, the transcriptional profiling analysis of 26 genes consisting of the entire ST biosynthetic gene cluster elucidated that the transcriptional regulation of LaeA is ST cluster-specific, as the genes adjacent to the ST cluster are not affected. To understand the effect of LaeA in the production of LOV, Bok and Keller introduced the partial LOV cluster of *A. terreus* into the *A. nidulans* Δ*laeA*, producing the LOV intermediate monocolin J (MONJ). The Δ*laeA*/LOV^+^ strain displayed reduced mRNA levels of both *lovE* (encoding a LOV-specific Zn2Cys6 transcription factor) and *lovC* (a LOV biosynthetic gene), and diminished MONJ production as well. Overexpression of *laeA* elevated the expression levels of genes required for PN, and LOV biosynthesis (*ipnA*, *lovE*, and *lovC*) and the production of corresponding secondary metabolites, while, interestingly, ST production was unaffected [87].

*A. flavus* LaeA exhibits mostly similar functions consistent with *A. nidulans* LaeA yet also plays distinct roles in growth and sexual development. The Δ*laeA* mutant lost the ability to produce many secondary metabolites including aflatoxin B1 and B2, cyclopiazonic acid, kojic acid (on YES media), and oryzachlorin (on DG18 media). On the other hand, overexpression of *laeA* led to the enhancement of some secondary metabolite productions, which are not typically observed in the WT. The sclerotia-specific metabolites Paspaline/paspalinine, aflatrem, and aflavinines were produced exclusively in the *OE::laeA*. This phenomenon is highly correlated with the increased sclerotia formation in the *OE::laeA* strain. Along with the effect in sclerotia production, LaeA affects seed colonization and lipase activity, closely related to the pathogenicity of *A. flavus* [89].

The opportunistic human pathogen, *A. fumigatus,* has been extensively studied due to its notorious virulence in humans constituting the majority of invasive aspergillosis in immunocompromised individuals [90]. Secondary metabolites including toxins and melanins have been recognized as virulence factors of invasive aspergillosis. Deletion of *laeA* suppressed not only the production of multiple secondary metabolites including the immunotoxin gliotoxin but also the expression of 13 SM biosynthetic gene clusters including *A. fumigatus*-specific mycotoxin clusters. The transcriptomic profiling analyses of WT, Δ*laeA*, and *C’laeA* strains revealed that LaeA positively controls the expression of up to 40% of major classes of SM biosynthetic genes such as nonribosomal peptide synthetases, polyketide synthases, and P450 monooxygenases [91]. Regarding the effect of LaeA on virulence, two *A. fumigatus* Δ*laeA* strains exhibited decreased virulence in the mouse pulmonary model, which is attributed to the reduced killing of neutrophil cells. These suggest a strong correlation between LaeA-mediated toxin production and invasive aspergillosis development by *A. fumigatus* [92,93]. 

## 4. NsdD, a Key Regulator of Conidiation and Sexual Development

The general life cycle of *Aspergillus* begins with vegetative growth. Spores start to form small germ tubes (germlings) and these tubes elongate in a highly-polarized manner resulting in hyphal growth. Under certain favorable conditions, Aspergilli initiate asexual or sexual reproductive processes. *Aspergillus* species primarily reproduce through asexual sporulation (conidiation), while few of them can reproduce via sexual development. 

Asexual development (conidiation) in Aspergilli takes place via orchestrated gene expression of multiple positive and negative regulators. In order to initiate conidiation, upstream activators induce the activation of *brlA*, which encodes C_2_H_2_ zinc finger TF [94,95], initiating the development of conidiophore and activating the expression of *abaA*. Then, the AbaA and WetA play crucial roles in conidiophore maturation during the middle and late stages of conidiation, respectively. This central regulatory pathway (*brlA* → *abaA* → *wetA*) acts in concert with other genes to control conidiation-specific gene expression, and determine the order of gene activation during development and spore maturation [96]. During this asexual stage in the lifecycle, *Aspergillus* species produce multicellular reproductive organs, termed conidiophores, each of which produces multiple chains of non-motile conidia.

**Figure 7:**
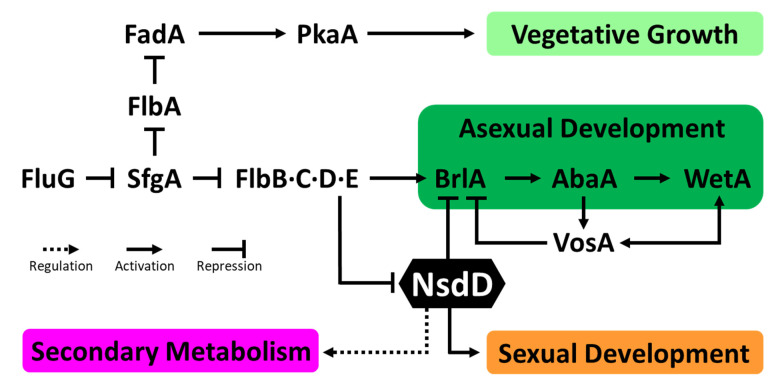
A genetic model for growth, developmental, and metabolic control in *Aspergillus*. An arrow with a solid line indicates a positive regulation (activation) in a relationship, an arrow with a dotted line indicates an unspecified regulation (can be activating or repressing), and a blunt-ended line indicates a repressive role in the relationship.

Previous studies revealed that the evolutionarily conserved GATA-type transcription factor (TF) NsdD acts as a key negative regulator of asexual development by downregulating the expression of *brlA* in *Aspergillus* (Figure 7) [14,97]. The NsdD directly binds to the promoter regions of the *brlA* gene and represses *brlA* expression in concert with another repressor VosA. The deletion of *nsdD* resulted in accelerated and precocious activation of conidiation; the mutant even produced asexual developmental organs under liquid submerged cultures where conidiation never takes place in the WT and exhibited the increased production of conidia on solid media. NsdD also plays a significant role in conidiophore morphogenesis. Deletion of *nsdD* resulted in abnormal hyphal branching during vegetative growth (data not shown). The *nsdD* null mutant in *A. flavus* displayed the formation of approximately 10 times shorter and 4 times smaller conidiophores that resemble closer to those of *A. nidulans* WT [14,98]. Moreover, NsdD regulates mycotoxin production including sterigmatocystin (ST) and aflatoxin (AF) in *A. nidulans* and *A. flavus*, respectively. Furthermore, previous studies have found that NsdD is required for proper sexual development. The deletion of *nsdD* resulted in no fruiting body formation, even under the sexual development promoting conditions. In contrast, overexpression of *nsdD* led to the increased formation of fruiting bodies and displayed resistance to certain inhibitory effects on sexual fruiting in *Aspergillus* [98,99,100]. Although the pleiotropic characteristics of NsdD regarding the development and metabolism of the genus *Aspergillus* have been well studied during the last two decades, the regulatory mechanism underlying how the single GATA-type TF NsdD governs all distinct aspects of fungal biology remains to be investigated.

## 5. Conclusions

In filamentous fungi *Aspergillus* species, biological and molecular processes are orchestrated by various temporal and spatial combinations of signal transduction mediators, transcription factors, regulators, and genes. In this review, among a large array of developmental and metabolic regulators identified in aspergilli, we focused on the most important upstream regulators that are known for their global impacts on *Aspergillus* biology, then summarized their specific roles on growth, development, and secondary metabolism. Due to their significances in fungal development and metabolism, a variety of studies have been conducted; thus, researchers now have a better understanding on their biological roles, but the detailed mechanisms of how individual regulators exert such diverse effects on different aspects of *Aspergillus* biology have yet to be unveiled. Genome-wide gene regulatory network analysis will be necessary to identify genes that are under the direct or indirect control of theses regulators, and to understand the relationship between direct and indirect target genes. The network study will provide valuable information upon which to understand the underlying regulatory mechanisms of main upstream regulators in governing development and metabolism of *Aspergillus* species. 

## Data Availability

Not applicable.
